# Supplementary material for: Hepatic Sinusoidal Obstruction Syndrome Secondary to Aplastic Anemia/Paroxysmal Nocturnal Hemoglobinuria Syndrome: A Rare Case
Source: Diagnostics (Basel). 2025 Oct 27;15(21):2712. doi: 10.3390/diagnostics15212712 (PMC12607371; doi:10.3390/diagnostics15212712)
Supplement: Supplementary file 1 [file diagnostics-15-02712-s001.zip › diagnostics-3893199-supplementary.pdf]

Supplementary Table 1. Contrasting Common Venous Thrombosis and HSOH in PNH

Comparison Dimension	PNH	HSOS
Primary Sites	venous system, especially in the hepatic and cerebral venous vessels	hepatic sinusoids, interlobular veins
Clinical Presentation	anemia, abdominal pain, dyspnea, fatigue, thrombosis	abdominal distension, loss of appetite, fatigue
Diagnostic Tools	ultrasonography, CT, MRI, flow cytometry	liver biopsy, CT
Mechanism	complement-mediated intravascular hemolysis, impaired nitric oxide bioavailability, activation of PNH platelets, and impairment of the fibrinolytic system	glutathione depletion, nitric oxide depletion, increased matrix metallo proteinases, increased vascular endothelial growth factor
Management	complement inhibitors, transfusions, iron supplementation, and anticoagulation	defibrotide, supportive care about fluid, diuretics, pain and complications, anticoagulants, glucocorticoids, interventional procedures
